# The *Botrytis cinerea* effector BcXYG1 suppresses immunity in *Fragaria vesca* by targeting FvBPL4 and FvACD11

**DOI:** 10.1093/hr/uhad251

**Published:** 2023-12-11

**Authors:** Liyao Su, Tian Zhang, Bin Yang, Yibo Bai, Wanping Fang, Jingsong Xiong, Zong-Ming (Max) Cheng

**Affiliations:** State Key Laboratory of Crop Genetics and Germplasm Enhancement, College of Horticulture, Nanjing Agricultural University, Nanjing 210095, China; State Key Laboratory of Crop Genetics and Germplasm Enhancement, College of Horticulture, Nanjing Agricultural University, Nanjing 210095, China; College of Horticulture, Nanjing Agricultural University, Nanjing 210095, China; State Key Laboratory of Crop Genetics and Germplasm Enhancement, College of Horticulture, Nanjing Agricultural University, Nanjing 210095, China; College of Horticulture, Nanjing Agricultural University, Nanjing 210095, China; State Key Laboratory of Crop Genetics and Germplasm Enhancement, College of Horticulture, Nanjing Agricultural University, Nanjing 210095, China; State Key Laboratory of Crop Genetics and Germplasm Enhancement, College of Horticulture, Nanjing Agricultural University, Nanjing 210095, China

## Abstract

*Botrytis cinerea* is one of the most destructive pathogens in strawberry cultivation. Successful infection by *B. cinerea* requires releasing a large number of effectors that interfere with the plant’s immune system. One of the effectors required by *B. cinerea* for optimal virulence is the secreted protein BcXYG1, which is thought to associate with proteins near the plasma membrane of the host plant to induce necrosis. However, the host proteins that associate with BcXYG1 at the plasma membrane are currently unknown. We found that BcXYG1 binds to FvBPL4 and FvACD11 at the plasma membrane. Both FvBPL4 and FvACD11 are negative regulators of plant immunity in strawberry. Our results demonstrate that degradation of FvBPL4 by BcXYG1 promotes disease resistance while stabilization of FvACD11 by BcXYG1 suppresses the immune response. These findings suggest that BcXYG1 suppresses plant immunity and promotes *B. cinerea* infection by regulating FvBPL4 and FvACD11 protein levels.

## Introduction

The battle between plants and pathogens is multi-layered. In response to the diverse means by which pathogens infect plants, a multi-tiered immune system has evolved in plants to repel these unwanted invaders [1]. The plant immune system consists of two layers of innate immunity: pattern-triggered immunity (PTI) and effector-triggered immunity (ETI). PTI is an early plant response to pathogen invasion. After pathogens breach the cell wall, pattern recognition receptors (PRRs) at the plasma membrane recognize conserved pathogen-associated molecular patterns (PAMPs) in the apoplastic space. Binding of PAMPs to the extracellular domain of PRRs initiates a signaling cascade that opens ion channels, produces a burst of reactive oxygen species (ROS), activates protein kinases, and results in the expression of genes involved in disease resistance [2, 3]. The PTI response culminates in broad-spectrum resistance against pathogens due to the conserved nature of PAMPs between pathogen races and even pathogens of different species. In contrast, ETI is typically a race-specific immune response that often culminates in programmed cell death. The ETI response is initiated by intracellular immune receptors called leucine-rich repeat receptors (NLRs) that use C-terminal leucine-rich repeat domains to recognize pathogen effectors and activate a signaling cascade leading to an immune response that is typically stronger than PTI [4, 5].

*Botrytis cinerea* is a necrotrophic fungal pathogen with a wide host range that is responsible for major economic losses worldwide [6–8]. *B. cinerea* is a widespread, filamentous, and necrotrophic fungus that infects more than 1000 plant species [9]. Disease progression typically occurs in two stages: spore germination on the leaf surface followed by rapid induction of host tissue necrosis. Rapidly growing hyphae secrete a cocktail of cell wall degrading enzymes to break down cellulose and pectin in the plant cell wall. Degradation of the cell wall releases sugars and proteins that can be metabolized by *B. cinerea* directly.

In addition to acquiring nutrients from cell wall components, *B. cinerea* also induces programmed cell death in host cells to acquire additional intracellular nutrients. Cell death induction is partly accomplished by the secretion of many necrosis-inducing proteins (NIPs) that degrade physical defense barriers during early colonization [10, 11]. Xyn11A and Xyl1 are two xylanase NIPs that contribute to the virulence of *B. cinerea* through their ability to induce necrosis [12, 13]. Multiple endopolygalacturonases secreted by *B. cinerea* can also induce plant necrosis through several modes of action. Among them, BcPG2 has a wide range of toxicity in different plant species [14, 15]. Additionally, BcIEB1 [16], BcNep1-like [17], and BcSpl1 [18] are secreted proteins that contribute to the full virulence of the *B. cinerea* during colonization. A recent study suggests that the secreted xylanase BcXYG1 may induce necrosis through activity at the host cell plasma membrane [19]. Unlike other effectors, BcXYG1 is unable to induce necrosis after heat treatment, indicating that the tertiary structure of BcXYG1 is critical to its function. However, the mechanism by which BcXYG1 induces necrosis and the host proteins it associates with at the plasma membrane are unknown but vital to understanding how NIPs such as BcXYG1 induce host cell death.

Accelerated cell death 11 (ACD11) regulates programmed cell death and defense responses in *Arabidopsis* by regulating the production of ROS [20, 21]. ACD11 interacts with BPA1 (binding partner of ACD11 1), PRA7, PRA8, and a protein of unknown function *in vivo* [22]. BPA1 and its BPL (binding partner of ACD11-like) homologs function as negative regulators of ROS homeostasis and defense responses [21]. ACD11 also associates with the cyclic E3 ligase XBAT35.2, which negatively regulates plant immunity by mediating the ubiquitin-dependent degradation of ACD11 [23]. In summary, the BPA/BPLs–ACD11 complex is an important component of plant immunity and is subject to multiple tiers of regulation.

**Figure 1:**
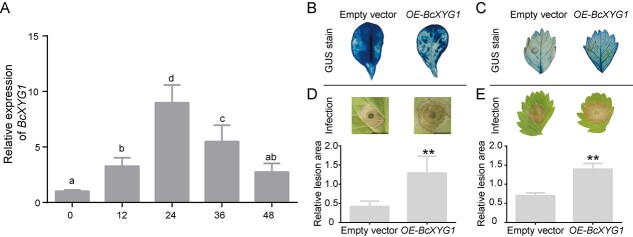
*BcXYG1* enhances susceptibility to *B. cinerea* in *N. tabacum* and *F. vesca*. **A** Relative expression of *BcXYG1* at 0, 12, 24, 36, and 48 hpi of *B. cinerea* spores on *F. vesca* leaves. Different letters indicate significant differences between means (LSD, *P* < 0.01). **B**, **C** BcXYG1-GUS and GUS were overexpressed in *N. tabacum* and *F. vesca* by leaf discs method. GUS staining was used to identify *N. tabacum* and *F. vesca* leaves overexpressing *BcXYG1*. **D**, **E** 15 µl drop of the *B. cinerea* spore suspension were inoculated on the leaves of transgenic *F. vesca* and *N. tabacum* (n = 3). The images were taken at 120 hpi after inoculated with B. cinerea spores. Lesion areas were calculated by ImageJ software and statistical significance were calculated by SPSS software. Asterisks indicate significant differences between means (LSD, ^**^P < 0.01).

Strawberries are one of the most economically important berries in the world as they are revered for their exceptional flavor and nutrients. In hot, humid environments, conidia of *B. cinerea* invade through stomata, hydathodes, or wounds [24]. However, *B. cinerea* presents a serious threat to strawberry cultivation due to crop loss in the field and post-harvest fruit loss. A better understanding of the strawberry immune response against *B. cinerea* infection is critical to developing more resistant strawberry varieties. In this study, we identify host targets of the BcXYG1 effector from *B. cinerea*. We found that BcXYG1 binds to both FvBPL4 and FvACD11 with opposing outcomes. FvBPL4 is degraded upon binding to BcXYG1 while FvACD11 accumulation is enhanced upon binding to BcXYG1. We also found that FvBPL4 stabilizes FvACD11. Our results suggest that the promoted degradation of FvBPL4 and stabilization of FvACD11 by BcXYG1, suppress the strawberry immune response to promote successful infection by *B. cinerea*.

## Results

### *Botrytis cinerea* secretes the xyloglucanase BcXYG1 to promote successful host colonization

BcXYG1 is a xyloglucanase secreted by *B. cinerea* that induces a strong cell death and immune response in host plants [19]. To study the dynamic changes in *BcXYG1* expression in *B. cinerea* during infection of *Fragaria vesca*, qPCR was performed on leaf tissue collected at 0, 12, 24, 36, and 48 h post-inoculation (hpi). A significant increase in *BcXYG1* expression occurred at 0–24 hpi, followed by a drop in expression from 24 to 48 hpi (Fig. 1A). This suggests that *BcXYG1* may play an important role in the initial stages of *B. cinerea* infection.

To further characterize the function of *BcXYG1* in plants, *BcXYG1* was overexpressed in *Nicotiana tabacum* (Fig. S1). Transgenic expression of *BcXYG1* was confirmed using GUS staining and qPCR (Fig. 1B, Supplementary Data Fig. S3A and B). Interestingly, leaf necrosis area in *N. tabacum* plants overexpressing *BcXYG1* was significantly larger than that in the control (Fig. 1D, Supplementary Data Fig. S2). We also obtained *F. vesca* plants transformed with *BcXYG1* and confirmed gene expression using GUS staining (Fig. 1C). Transgenic *F. vesca* plants overexpressing *BcXYG1* were also more susceptible to *B. cinerea* compared with the negative control plants (Fig. 1E). These results suggest that *BcXYG1* can promote *B. cinerea* infection of *N. tabacum* and *F. vesca*.

### BcXYG1 interacts with FvBPL4 at the plasma membrane

**Figure 2:**
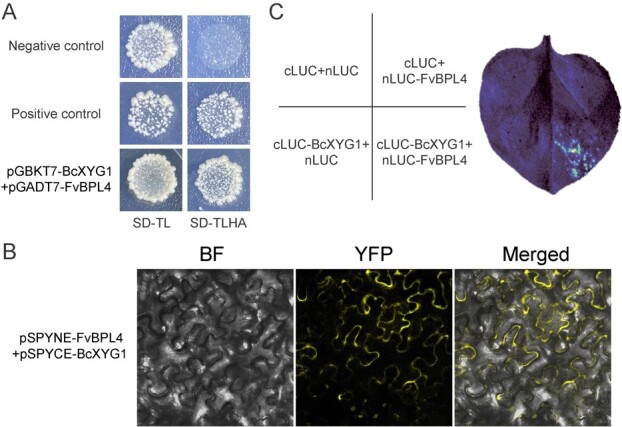
BcXYG1 interacts with FvBPL4 at the plasma membrane. **A** Verification of the interaction between BcXYG1 and FvBPL4 by yeast two-hybrid analysis. **B** Verification of the interaction between BcXYG1 and FvBPL4 by BiFC analysis in *N. benthamiana* leaves. The vectors pSPYNE-FvBPL4 and pSPYCE-BcXYG1 were transiently co-expressed in *N. benthamiana* leaf. Bright-field (BF) and YFP fluorescence images were taken using a confocal laser-scanning microscope (514 nm excitation) and merged. A more detailed picture is shown in Supplementary Data Fig. S5. **C** Verification of the interaction between BcXYG1 and FvBPL4 by LCI analysis. The vectors pCAMBIA1300-FvBPL4-NLuc and pCAMBIA1300-BcXYG1-CLuc were transiently co-expressed in *N. benthamiana* leaf.

To identify potential target proteins of BcXYG1 in *F. vesca*, we used BcXYG1 as a bait to screen an *F. vesca* cDNA library in a yeast two-hybrid assay. We identified FvBPL4 as a potential binding partner of BcXYG1 and confirmed the interaction in yeast (Fig. 2A). To further confirm the interaction, we performed a bimolecular fluorescence complementation (BiFC) assay in *Nicotiana benthamiana* leaves. The results showed that co-expression of *FvBPL4-nYFP* and *BcXYG1-cYFP* generates a yellow fluorescent signal indicative of a successful protein–protein interaction (Fig. 2B). A luciferase complementation imaging (LCI) assay in *N. benthamiana* leaves further confirmed the interaction, since a luminescent signal was only generated when *FvBPL4-nLUC* and *BcXYG1-cLUC* were co-expressed but luminescence was not generated in any of the negative controls (Fig. 2C). The subcellular localization of FvBPL4-GFP appeared near the plasma membrane and within the nucleus in *N. benthamiana* leaf cells, whereas BcXYG1-GFP was found exclusively at the plasma membrane (Supplementary Data Fig. S4). Together, these results suggest that FvBPL4 and BcXYG1 associate near the plasma membrane.

### Overexpression of *FvBPL4* promotes *B. cinerea* infection

**Figure 3:**
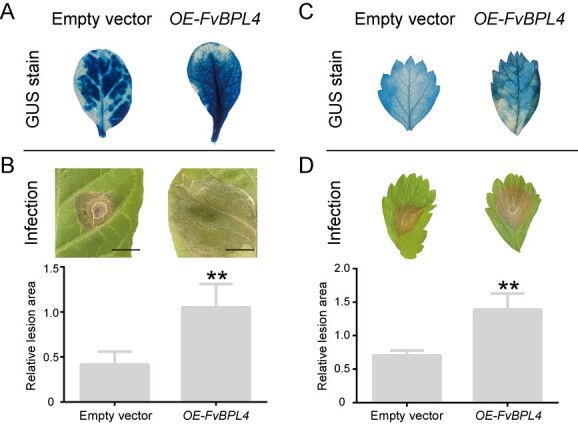
Overexpression of *FvBPL4* promotes *B. cinerea* infection in *N. tabacum* and *F. vesca*. **A**, **C** FvBPL4-GUS and GUS were overexpressed in *N. tabacum* and *F. vesca* by leaf discs method. Transgenic plants were identified using GUS staining. **B**, **D** Leaves of *N. tabacum* and *F. vesca* overexpressing *FvBPL4* were inoculated with 15 µl drop *B. cinerea* spores (n=3) and images of infected leaves were taken after 5 days to assess necrosis. Lesion areas on *N. tabacum* and *F. vesca* leaves were measured at 120 hpi. Asterisks indicate significant differences between means (LSD, ^**^P < 0.01).

To characterize the role played by FvBPL4 in *B. cinerea* infection, we generated stable transgenic *N. tabacum* and *F. vesca* lines overexpressing *FvBPL4* (Supplementary Data Fig. S1). Transgene expression in the seedlings of both plants was confirmed using GUS staining and qPCR (Fig. 3A and C, Supplementary Data Fig. S3C and D). We inoculated *B. cinerea* spores on the leaves of transgenic plants and assessed the area of necrosis at 5 days post-inoculation. Leaves from both *N. tabacum* and *F. vesca* overexpressing *FvBPL4* exhibited greater tissue necrosis than plants transformed with an empty vector (Fig. 3B and D, Supplementary Data Fig. S2). These results suggest that *FvBPL4* promotes *B. cinerea* virulence in *N. tabacum* and *F. vesca*.

### FvBPL4 and BcXYG1 bind to FvACD11 at the plasma membrane

**Figure 4:**
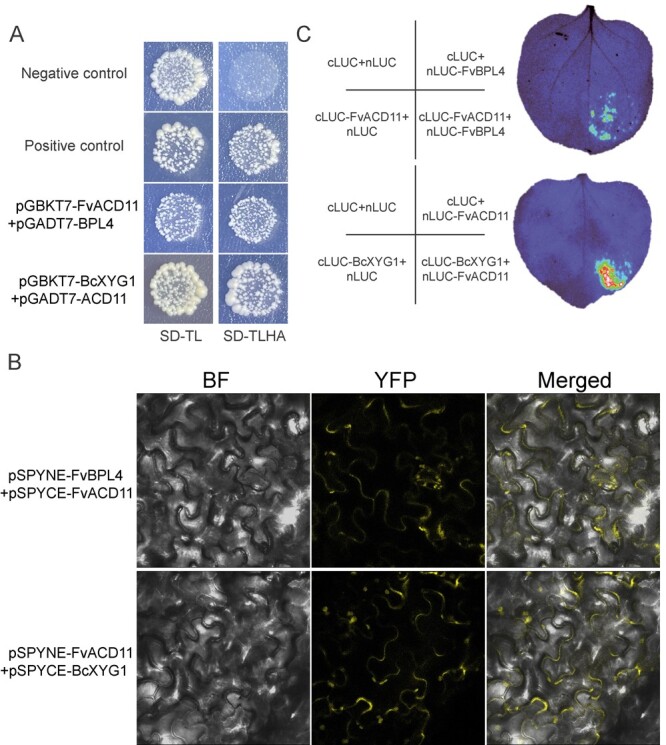
FvACD11 binds to FvBPL4 and BcXYG1 in yeast and *N. benthamiana*. **A** Yeast two-hybrid assay demonstrating that FvACD11 can interact with BcXYG1 and FvBPL4. **B** BiFC assay demonstrating that FvACD11 can interact with BcXYG1 and FvBPL4 in *N. benthamiana* epidermal cells. The vectors pSPYNE-FvBPL4/pSPYCE-FVACD11 and pSPYNE-FvACD11/pSPYCE-BcXYG1were transiently co-expressed in *N. benthamiana* leaf. Bright-field (BF) and YFP fluorescence images were taken using a confocal laser-scanning microscope (514 nm excitation) and merged. A more detailed picture is shown in Supplementary Data Fig. S5. **C** LCI assay demonstrating that FvACD11 can interact with BcXYG1 and FvBPL4 in *N. benthamiana* leaves. The vectors pCAMBIA1300-FvBPL4-NLuc/pCAMBIA1300-FvACD11-CLuc and pCAMBIA1300-FvACD11-NLuc/pCAMBIA1300-BcXYG1-CLuc were transiently co-expressed in *N. benthamiana* leaf.

BPA and BPL family members from other species are known to associate with ACD11. The origins of this ancient protein complex can be traced back to a time before plants had colonized the land [21, 26]. We sought to determine if FvBPL4 could associate with FvACD11. A yeast two-hybrid assay demonstrated that FvBPL4 and FvACD11 associate in yeast while BiFC and LCI assays demonstrated that FvBPL4 and FvACD11 associate in *N. benthamiana* (Fig. 4A–C). Using the same assays, we also demonstrated that BcXYG1 and FvACD11 associate in yeast and *N. benthamiana* (Fig. 4). The subcellular localization of FvACD11 appears to be primarily at the plasma membrane and nucleus of *N. benthamiana* cells (Supplementary Data Fig. S4). Together, these results suggest that both FvBPL4 and BcXYG1 associate with FvACD11 and that FvACD11 is one of the targets of BcXYG1 at the plasma membrane.

### Overexpression of *FvACD11* promotes *B. cinerea* infection

**Figure 5:**
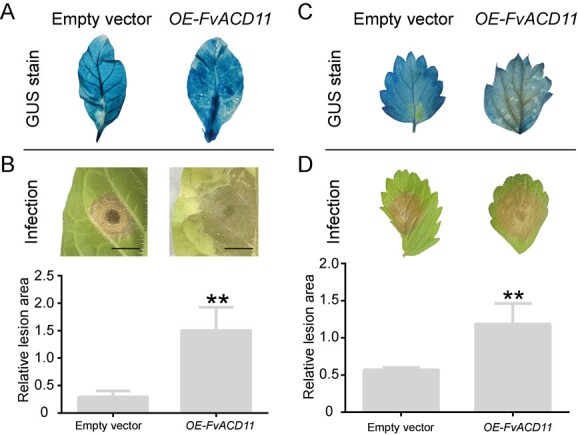
Overexpression of *FvACD11* promotes *B. cinerea* infection in *N. tabacum* and *F. vesca*. **A**, **C** FvACD11-GUS and GUS were overexpressed in *N. tabacum* and *F. vesca* by leaf discs method. Transgenic plants were identified using GUS staining. **B**, **D** Leaves of *N. tabacum* and *F. vesca* overexpressing *FvACD11* were inoculated with 15 µl drop*B. cinerea* spores (n=3) and images of infected leaves were taken after 5 days to assess necrosis. Lesion areas on *N. tabacum* and *F. vesca* leaves were measured at 120 hpi. Asterisks indicate significant differences between means (LSD, ^**^P < 0.01).

To characterize the effect of *FvACD11* in *B. cinerea* infection, we produced stable transgenic *N. tabacum* and *F. vesca* lines overexpressing *FvACD11* (Supplementary Data Fig. S1). We confirmed the transgene expression in the seedlings of both plants using GUS staining and qPCR (Fig. 5A and C, Supplementary Data Fig. S3E and F). We inoculated the leaves of transgenic plants with *B. cinerea* spores and assessed the area of necrosis at 5 days post-inoculation. Leaves of *N. tabacum* and *F. vesca* overexpressing *FvACD11* exhibited greater tissue necrosis compared with plants transformed with the empty vector (Fig. 5B and D, Supplementary Data Fig. S2). These results suggest that FvACD11 promotes *B. cinerea* virulence in *N. tabacum* and *F. vesca*.

### BcXYG1 promotes degradation of FvBPL4 and stabilization of FvACD11

**Figure 6:**
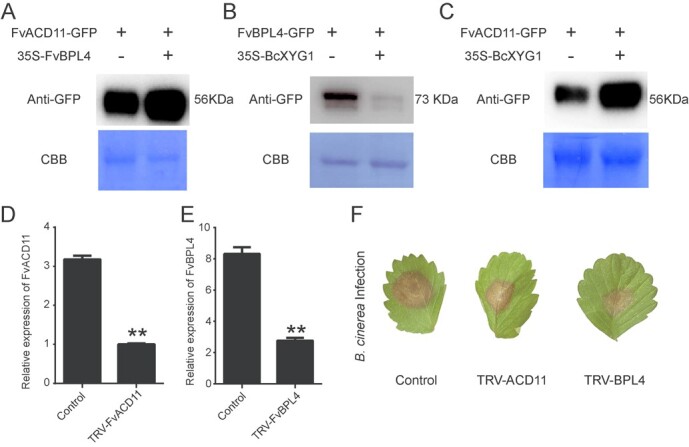
BcXYG1 promotes FvBPL4 degradation and FvACD11 accumulation. **A**–**C** Total proteins were extracted from *N. benthamiana* leaves expressing (**A**) *FvACD11-GFP/35S-FvBPL4*, (**B**) *FvBPL4-GFP/35S-BcXYG1* and (**C**) *FvACD11-GFP/ /35S-BcXYG1*. GFP-tagged proteins were detected and quantified using a western blot. CBB, Bradford method. The relative expression of (**D**) *FvBPL4* and (**E**) *FvACD11* in *FvBPL4*-silenced and *FvACD11*-silenced *F. vesca* leaves. **F***FvBPL4*-silenced, *FvACD11*-silenced, and control *F. vesca* leaves were inoculated with 15 µl drop *B. cinerea* spores and images were taken after 120 h.

Previous studies have shown that BPL4 homologs in other plant species can promote the accumulation of ACD11, which is consistent with our results (Fig. 6A) [21]. Studies have also demonstrated that pathogen effectors have evolved to promote the degradation or accumulation of BPL4, ACD11, and BPA1 homologs in other plant species. The *Phytophthora capsici* effector RxLR207 promotes infection by targeting AtBPL4 and AtACD11 for degradation [21], whereas the *Plasmopara viticola* effector RxLR50253 supports pathogen infection by promoting the accumulation of VpBPA1 [25]. To better understand the regulatory role played by BcXYG1 on FvBPL4 and FvACD11, we co-expressed *BcXYG1* with *FvBPL4-GFP* and *FvACD11-GFP* in *N. benthamiana* leaves and performed a western blot to assess protein accumulation. The results demonstrated that FvBPL4 accumulation was reduced upon *BcXYG1* coexpression whereas FvACD11 accumulated to greater levels when *BcXYG1* was coexpressed (Fig. 6B and C). This novel finding supports a model in which BcXYG1 differentially regulates FvBPL4 and FvACD11 to promote pathogenesis.

**Figure 7:**
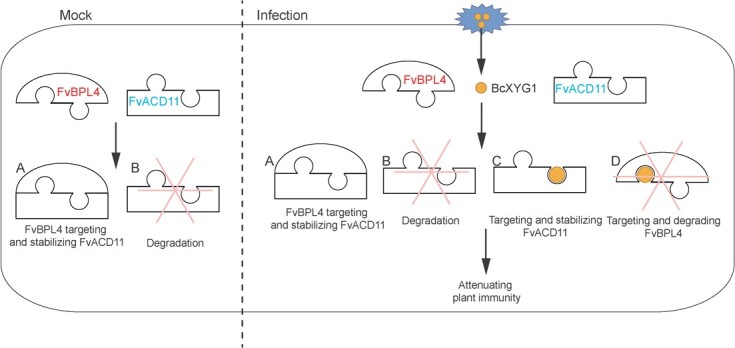
The proposed model explains how BcXYG1 acts on FvBPL4 and FvACD11 to promote *B. cinerea* infection. In the absence of the pathogen (A), FvBPL4 binds to FvACD11, resulting in greater accumulation of FvACD11 protein. Upon infection by *B. cinerea* (B), BcXYG1 is secreted and binds to FvACD11 at the plasma cell membrane. This interaction inhibits FvACD11 degradation (C). Concurrently, BcXYG1 binds to FvBPL4 at the plasma cell membrane and promotes its degradation (D).

To further understand the functions of *FvBPL4* and *FvACD11*, we used virus-induced gene silencing (VIGS) to silence each of these genes in *F. vesca* leaves (Fig. 6D and E). We found that silencing *FvBPL4* or *FvACD11* in *F. vesca* leaves resulted in enhanced resistance against *B. cinerea* (Fig. 6F). Enhanced resistance against *B. cinerea* in *FvBPL4*-silenced plants supports our finding that overexpression of *FvBPL4* leads to greater susceptibility (Fig. 3B and D). However, it appears to contradict our finding that coexpression of *BcXYG1* with *FvBPL4* leads to reduced FvBPL4 protein accumulation, which we would expect to result in enhanced resistance (Fig. 6C). We hypothesize that BcXYG1 associates with BPA/BPLs and ACD11 separately rather than the BPA/BPLs–ACD11 complex. Enhanced immunity from the BPA/BPLs–ACD11 complex is possibly obtained by the enhanced stability of ACD11 in the complex (Fig. 7).

## Discussion

As a pathogen with a necrotrophic lifestyle, *B. cinerea* must evade an immune response from living plant cells and quickly induce cell death to obtain nutrients from its host. Studies on plant pathogens coping with this dilemma suggest that these pathogens may have an autotrophic process that enables them to subvert host defenses [28–30]. However, this autotrophic stage was not observed in some studies [19]. *Botrytis cinerea* releases a large repertoire of cell wall degrading enzymes early in the infection process, and a large number of these are NIPs that serve an essential role in successful infection [12, 13, 15, 16, 19]. BcXYG1 is an NIP secreted early during infection by *B. cinerea* that induces severe plant necrosis [19]. When we expressed *BcXYG1* in *N. tabacum* and *F. vesca*, the necrosis area induced by *B. cinerea* increased significantly (Fig. 1). These results suggest that BcXYG1 functions as a *bona fide* necrosis-inducing effector secreted by *B. cinerea* to promote successful pathogenesis.

Unlike other NIPs, BcXYG1 is unable to induce necrosis after undergoing a heat treatment, suggesting that a stable tertiary structure is essential for its activity [19]. A previous study suggests that the necrotic activity of BcXYG1 may be mediated by the RLP–SOBIR1–BAK1 complex, and the target of BcXYG1 could be located at the plasma membrane [19]. We screened BcXYG1 against an *F. vesca* cDNA library and identified FvBPL4 and FvACD11 as interacting partners (Figs 2A and4A). The interactions between BcXYG1, FvBPL4, and FvACD11 were confirmed using BiFC and LCI assays (Figs 2 and4). We demonstrated that *FvBPL4* and *FvACD11* negatively regulate resistance against *B. cinerea* in plants(Figs 3–6).

Sphingolipids and their metabolites serve as secondary messengers that play an important role in regulating the cell cycle in plants [31, 32]. The *Arabidopsis acd11* and *acd5* mutants accumulate high levels of sphingolipids that ultimately lead to programmed cell death [20, 27, 33]. ACD11 has been reported to interact with all BPA1 and BPL homologs in *Arabidopsis* [21], and evolutionary analyses have revealed that they are present in all terrestrial plants. This suggests that the immunological role played by these proteins predates land plants [26]. We demonstrated that both FvBPL4 and BcXYG1 can interact with FvACD11, and this association takes place at the plasma membrane (Fig. 4). These results support previous findings by Li *et al*. [21] demonstrating that the *P. capsici* effector RxLR207 promotes plant immunity through degradation of AtBPA1, AtBPL1/2/4, and AtACD11. Additionally, the *P. viticola* effector RxLR50253 can bind and stabilize VpBPA1 to promote pathogen infection [25]. Taken together, these results suggest that BPA, BPLs, and ACD11 are negative regulators of plant disease resistance [21, 25].

In this study, we demonstrated that BcXYG1 promotes the degradation of FvBPL4, but enhances the stability of FvACD11 (Figs 6 and7). We suggest that effector BcXYG1 targets FvBPL4 and FvACD11 through a novel mechanism. Silencing of *FvBPL4* or *FvACD11* in *F. vesca* leaves resulted in reduced susceptibility to *B. cinerea* (Fig. 6). We also show that FvBPL4 can stabilize FvACD11 (Fig. 6). These results suggest that the functions of *BPL4* and *ACD11* are conserved in plants [21, 25]. BcXYG1 degrades FvBPL4 to promote plant immunity but stabilizes FvACD11 to suppress plant immunity. We speculate that BcXYG1 acts on the BPA/BPLs and ACD11 alone instead of the BPA/BPLs–ACD11 complex, and the resistance conferred by the BPA/BPLs–ACD11 complex is disrupted by regulating the degradation and stabilization of ACD11 (Fig. 7).

In summary, we identified two *F. vesca* targets of the *B. cinerea* NIP effector BcXYG1: FvBPL4 and FvACD11. FvBPL4 and FvACD11 can form a complex that regulates plant resistance to fungal pathogens. BcXYG1 targets the BPA/BPLs–ACD11 complex through a novel mechanism by degrading FvBPL4 but stabilizing FvACD11, which leads to compromised immunity. This study provides new insights into the mechanisms of recognition and regulation of effector complexes containing FvBPL4 and FvACD11.

## Materials and methods

### Vector construction

PrimeSTAR HS DNA Polymerase (TaKaRa, Beijing, China) was used to amplify *BcXYG1*, *FvBPL4*, and *FvACD11* from mixed cDNA samples of strawberry (*F. vesca*) leaves infected with *B. cinerea* and collected at 24, 36, and 48 hpi. *FvBPL4* and *FvACD11* were subcloned into pGADT7, pSPYNE, pCAMBIA1300-CLuc, pCAMBIA1302, and pCAMBIA1301 vectors by seamless cloning while *BcXYG1* and *FvACD11* were subcloned into pGBKT7, pSPYCE, pCAMBIA1300-CLuc, pCAMBIA1302, and pCAMBIA1301 vectors using seamless cloning. Constructs used for VIGS were created by subcloning *FvBPL4* and *FvACD11* into the pTRV2 vector. All vectors used in this study were obtained from the State Key Laboratory of Crop Genetics and Germplasm Enhancement (College of Horticulture, Nanjing Agricultural University). All primers used for PCR can be found in Supplementary Data Table S1.

### *Fragaria vesca* and *N. tabacum* transgenic plants

The recombinant plasmids pCAMBIA1301-BcXYG1-GUS, pCAMBIA1301-FvBPL4-GUS, and pCAMBIA1301-FvACD11-GUS were introduced into *Agrobacterium tumefaciens* strain GV3101 using the heat-shock transformation method. Leaves from sterile histoculture seedlings of *F. vesca* and *N. tabacum* were obtained from the State Key Laboratory of Crop Genetics and Germplasm Enhancement (College of Horticulture, Nanjing Agricultural University, Nanjing, China). Using the leaf disc method, we obtained transgenic *F. vesca* and *N. tabacum* plants expressing *BcXYG1*, *FvBPL4*, and *FvACD11*. Transgenic plants were grown in a greenhouse at 25°C with 16 h of light and 8 h of darkness. Tissue was collected for RT–qPCR to assess overexpressing efficiency.

### β-Glucuronidase staining

X-Gluc powder (Yeasen, Shanghai, China) was dissolved in N,N-Dimethylformamide (DMF) solution to a final concentration of 40 mM and stored in a freezer at −80°C. GUS staining solution was prepared by combining the following: 1 ml of 0.5 M phosphate buffer, 0.1 ml of 10% Triton X-100, 0.2 ml of 100 mM K_3_Fe(CN)_6_, 0.2 ml of 100 mM K_4_[Fe(CN)_6_]·3H_2_O, 0.2 ml of 0.5 M EDTA2Na, 8.1 ml of ddH2O, and 0.2 ml of 40 mM X-Gluc. The leaves of transgenic plants were completely submerged in the staining solution and incubated overnight at 37°C. The next day, leaves were transferred to 95% ethanol until all chlorophyll was removed. The leaves were then observed and photographed.

### Yeast two-hybrid assays

BcXYG1 was used as bait to screen against a cDNA library created from *F. vesca* tissue. Potential interacting partners (FvBPL4 and FvACD11) identified from the initial screen were confirmed by co-transforming pGBKT7-BcXYG1 with individual prey candidates into the Y2H Gold yeast strain. We cloned *FvBPL4* and *FvACD11* into the pGADT7 vector and co-transformed the Y2H Gold yeast strain with pGBKT7-BcXYG1, respectively. In addition, we further cloned *FvACD11* into the pGBKT7 vector and co-transformed Y2H Gold yeast strain with pGADT7-FvBPL4. All co-transformed strains were plated on SD-Trp-Leu medium (Coolaber, Beijing, China). After 3 days of growth, single colonies were picked, suspended in sterile water, and adjusted to an OD_600_ of 0.05. Five microliters of each suspension was inoculated in SD-Trp-Leu-His-Ade medium (Coolaber, Beijing, China) and incubated at 30°C. After three days, we observed the growth status of yeast cells transformed in different combinations.

### Confocal microscopy

GV3101 *Agrobacterium* strains harboring pCAMBIA1302 (empty vector), pCAMBIA1302-FvBPL4-GFP, pCAMBIA1302-FvACD11-GFP, or pCAMBIA1302-BcXYG1-GFP were infiltrated into *N. benthamiana* leaves and incubated for 24 h in the dark followed by 48 h in the light. The subcellular localizations of FvBPL4-GFP, FvACD11-GFP, and BcXYG1-GFP were observed. Based on the experimental results of yeast two-hybrid assays, we cloned *FvBPL4* and *FvACD11* into pSPYNE vector and *FvACD11* and *BcXYG1* into pSPYCE vector. Subsequently, *Agrobacterium* containing recombinant plasmids was further obtained by heat-shock transformation method. *Agrobacterium* strains harboring the split-YFP constructs pSPYNE, pSPYCE, pSPYNE-FvBPL4, pSPYCE-FvACD11, or pSPYCE-BcXYG1 were co-infiltrated into *N. benthamiana* leaves and fluorescence from YFP was observed after 3 days. The microscope used for observation was a Zeiss LSM 800 with excitation wavelengths of 488 and 514 nm for GFP and YFP, respectively.

### Luciferase complementation imaging assay

Based on the experimental results of yeast two-hybrid assays, we cloned *FvBPL4 *and *FvACD11 *into pCAMBIA1300-NLuc vector and *FvACD11 *and *BcXYG1 *into pCAMBIA1300-CLuc vector. GV3101 strains harboring the pCAMBIA1300-NLuc, pCAMBIA1300-CLuc, pCAMBIA1300-FvBPL4-NLuc, pCAMBIA1300-FvACD11-NLuc, pCAMBIA1300-FvACD11-CLuc, or pCAMBIA1300-BcXYG1-Cluc plasmids were co-infiltrated into *N. benthamiana* leaves. Plants were incubated for 24 h in the dark followed by 48 h in the light. After the light incubation period, a 15 mM solution of potassium fluorescein (Coolaber, Beijing, China) was sprayed on the leaves. A CCD camera (Tanon 5200, Shanghai, China) was used to capture luminescence generated by luciferase activity with three biological replicates.

### Virus-induced gene silencing assay

To silence *FvACD11 *and *FvBPL4 *in *F. vesca*, the optimal silencing fragments of *FvACD11 *and *FvBPL4 *were predicted via an online website (https://vigs.solgenomics.net/), and the predicted fragments were cloned into the TRV2 vector. Suspensions of GV3101 strains harboring the VIGS plasmids TRV1, TRV2, TRV2-FvACD11, or TRV2-FvBPL4 were adjusted to an OD_600_ of 0.5. Strains were mixed in a 1:1 ratio and co-infiltrated into *F. vesca* leaves using vacuum infiltration. Infiltrated leaves were incubated in a moist Petri dish at 25°C. After 3 days, tissue was collected for RT–qPCR to assess silencing efficiency.

### Culture and inoculation of *B. cinerea*

*Botrytis cinerea* was cultured in complete medium for 2 weeks at 25°C in the dark. Spores were suspended in salt meat broth medium and adjusted to a final concentration of 1 × 10^6^ spores/ml. Leaves of *F. vesca* and *N. tabacum* plants were inoculated with a 15-μl drop of the spore suspension. Three biological replicates were set up for each experiment.

### Total RNA extraction and qPCR

Total RNA was extracted from all plants using the Plant Total RNA Isolation Kit (Foregene, Chengdu, China). PrimeScript™ RT (TaKaRa, Beijing, China) was used to synthesize the cDNA used in qPCR according to the manufacturer’s instructions. qPCR was performed using a LightCycler 480 and Hieff Unicon^®^ Universal Blue qPCR SYBR Green Master Mix (Yeasen, Shanghai, China). For qPCR analysis we used three biological replicates and three technical replicates. *B05.10 hypothetical protein* and *FvActin* were used as reference genes for *B. cinerea* and *F. vesca*, respectively. All primers used are listed in Supplementary Data Table S1.

### Western blot

To understand the relationship between BcXYG1, FvBPL4 and FvACD11, we used the pCAMBIA1302-FvBPL4-GFP, pCAMBIA1302-FvACD11-GFP and pCAMBIA1302-BcXYG1-GFP vectors constructed above as well as the pCAMBIA1301- BcXYG1 and pCAMBIA1301-FvBPL4 vectors. We injected tobacco leaves with pCAMBIA1302-FvACD11-GFP + pCAMBIA1301-FvBPL4, pCAMBIA1302-FvBPL4-GFP + pCAMBIA1301-BcXYG1 and pCAMBIA1302-FvACD11-GFP + pCAMBIA1301-BcXYG1 combinations. Three days after infiltrating *N. benthamiana* leaves with *Agrobacterium*, 0.1 g of leaf tissue was collected and ground to a fine powder in liquid nitrogen. Total protein was extracted using a Plant Total Protein Extraction Kit (Yeasen, Shanghai, China) according to the manufacturer’s instructions. An anti-GFP primary antibody was used to detect GFP fusion proteins. A goat anti-mouse antibody was used as the secondary antibody. Three biological replicates were set up for each experiment.

## Supplementary Material

Web_Material_uhad251
