# Supplementary material for: The Botrytis cinerea effector BcXYG1 suppresses immunity in Fragaria vesca by targeting FvBPL4 and FvACD11
Source: Hortic Res. 2023 Dec 11;11(1):uhad251. doi: 10.1093/hr/uhad251 (PMC10831327; doi:10.1093/hr/uhad251)
Supplement: Web_Material_uhad251 [file web_material_uhad251.zip › Supplementary figures.docx]

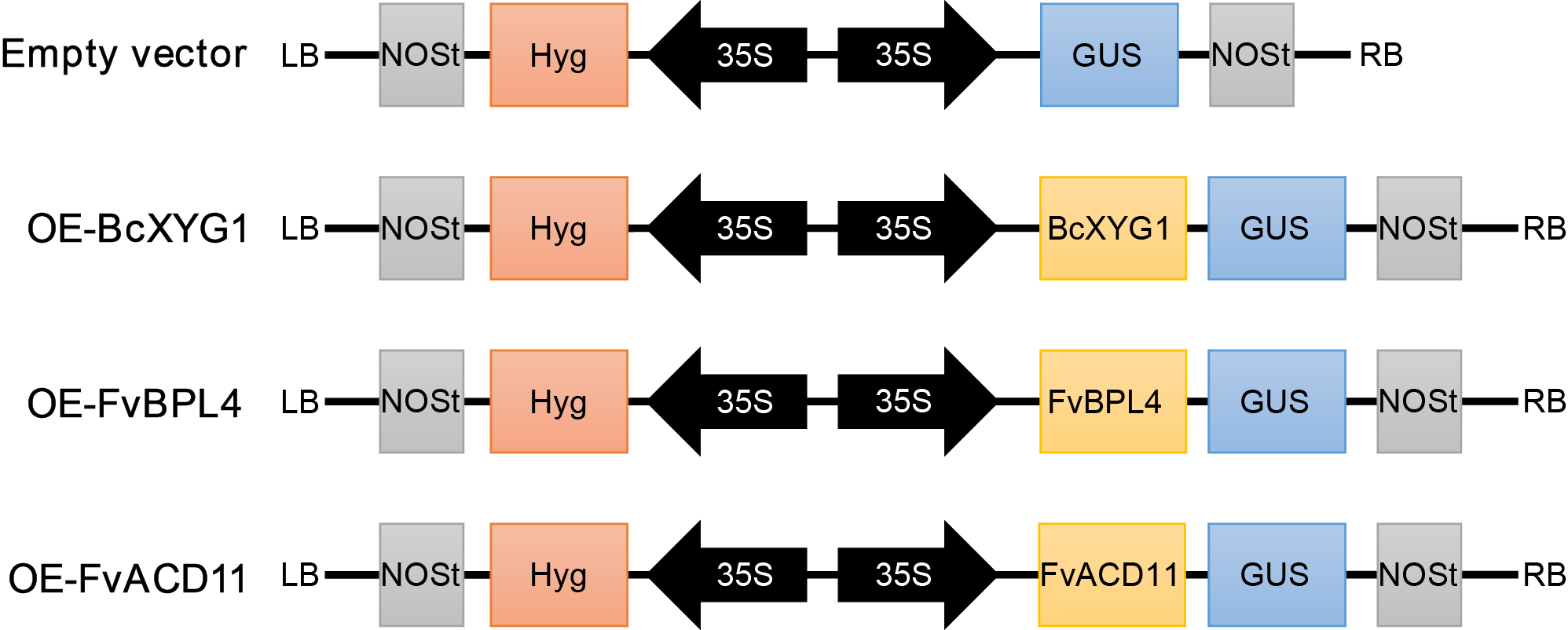


Supplementary figure 1. The strategy for overexpressing *BcXYG1*, *FvBPL4* and *FvACD11* vector construction.


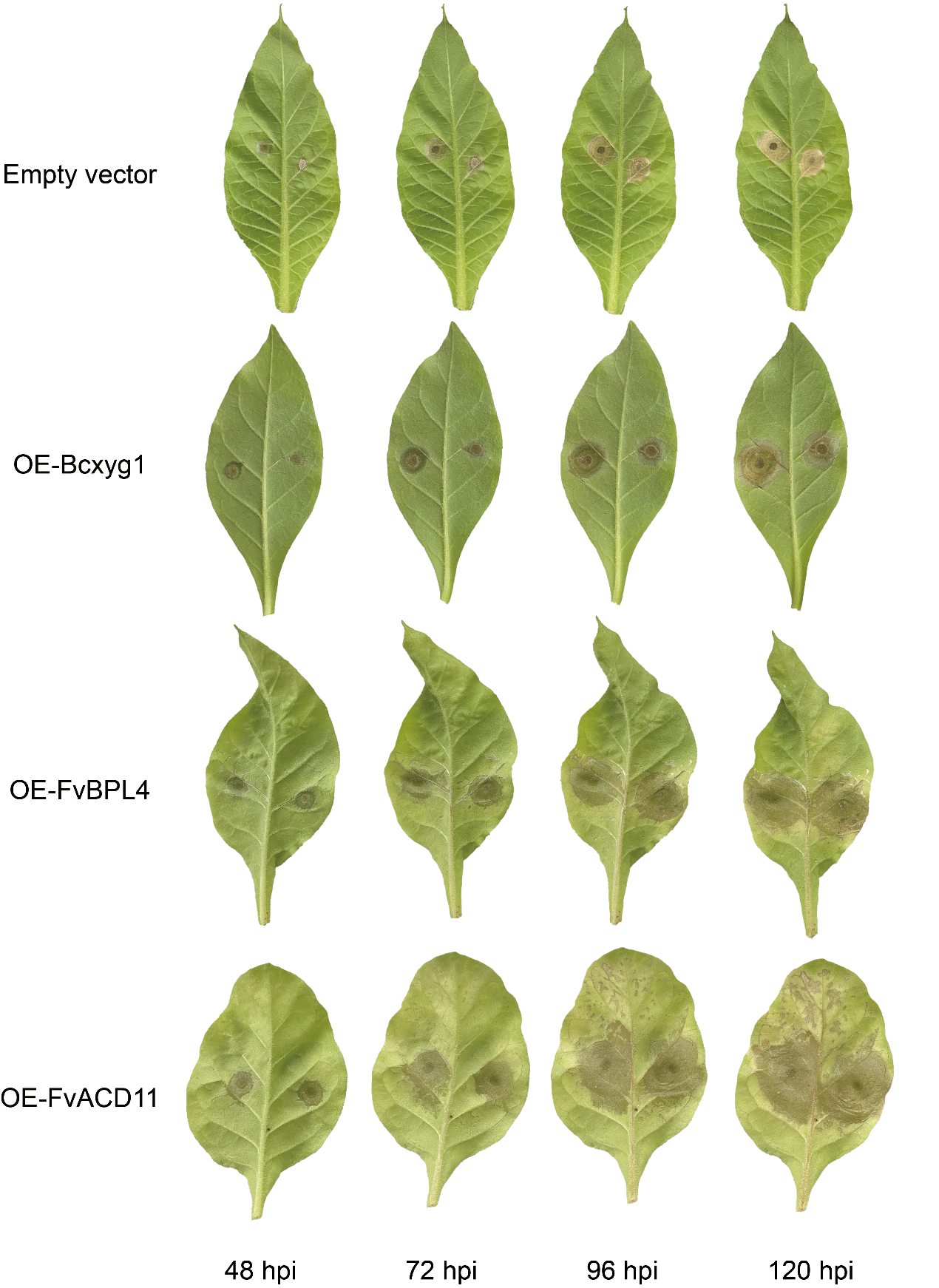


Supplementary figure 2. Leaves of *Nicotiana tabacum* overexpressing *BcXYG1*, *FvBPL4* and *FvACD11* were inoculated with *B. cinerea* spores.


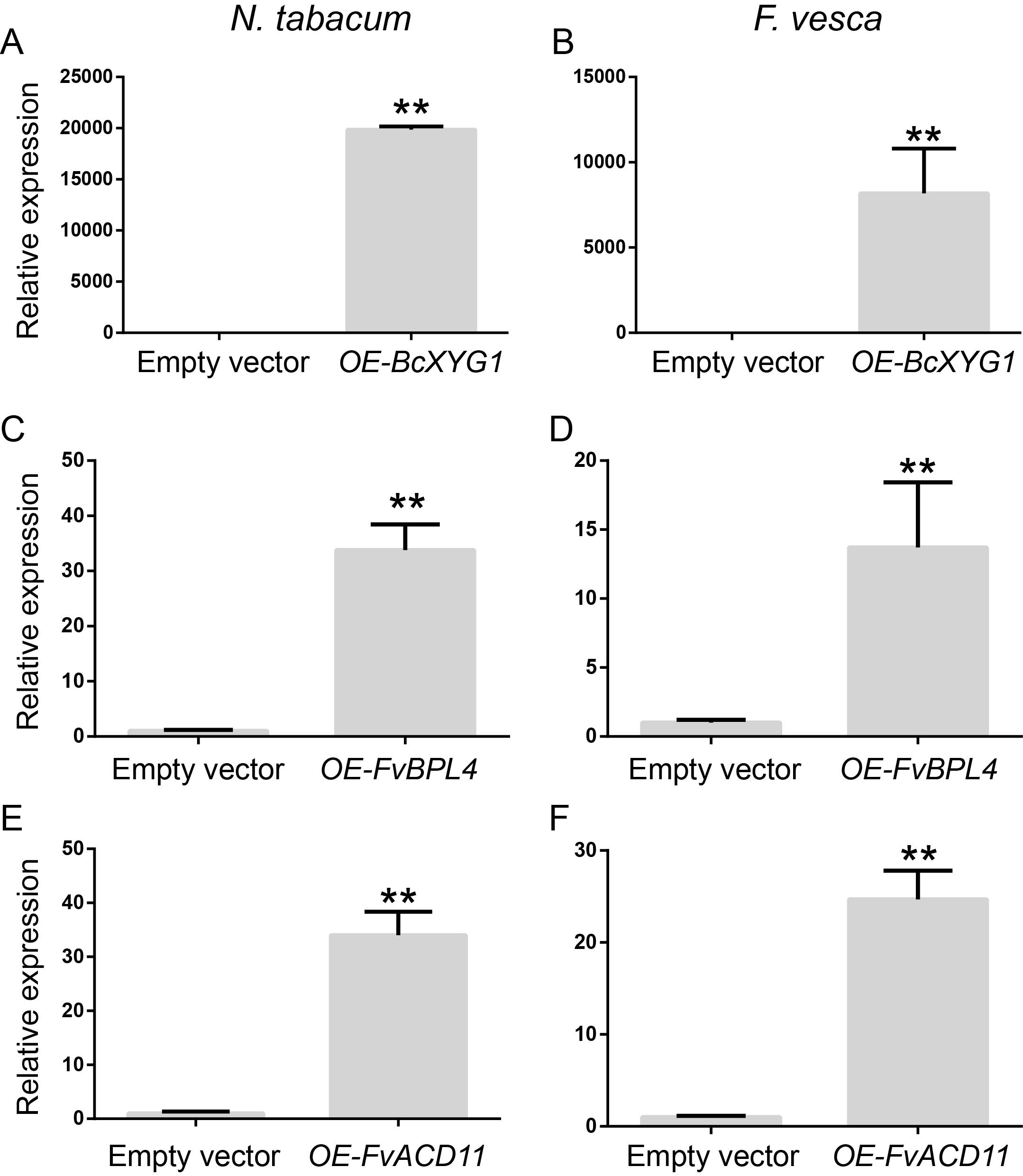


Supplementary figure 3. Expression of *BcXYG1*, *FvBPL4* and FvACD11 in *35S: BcXYG1, 35S: FvBPL4* and *35S:FvACD11* transgenic *N.tabacum* (A, C and E) and *F.vesca* (B, D and F).


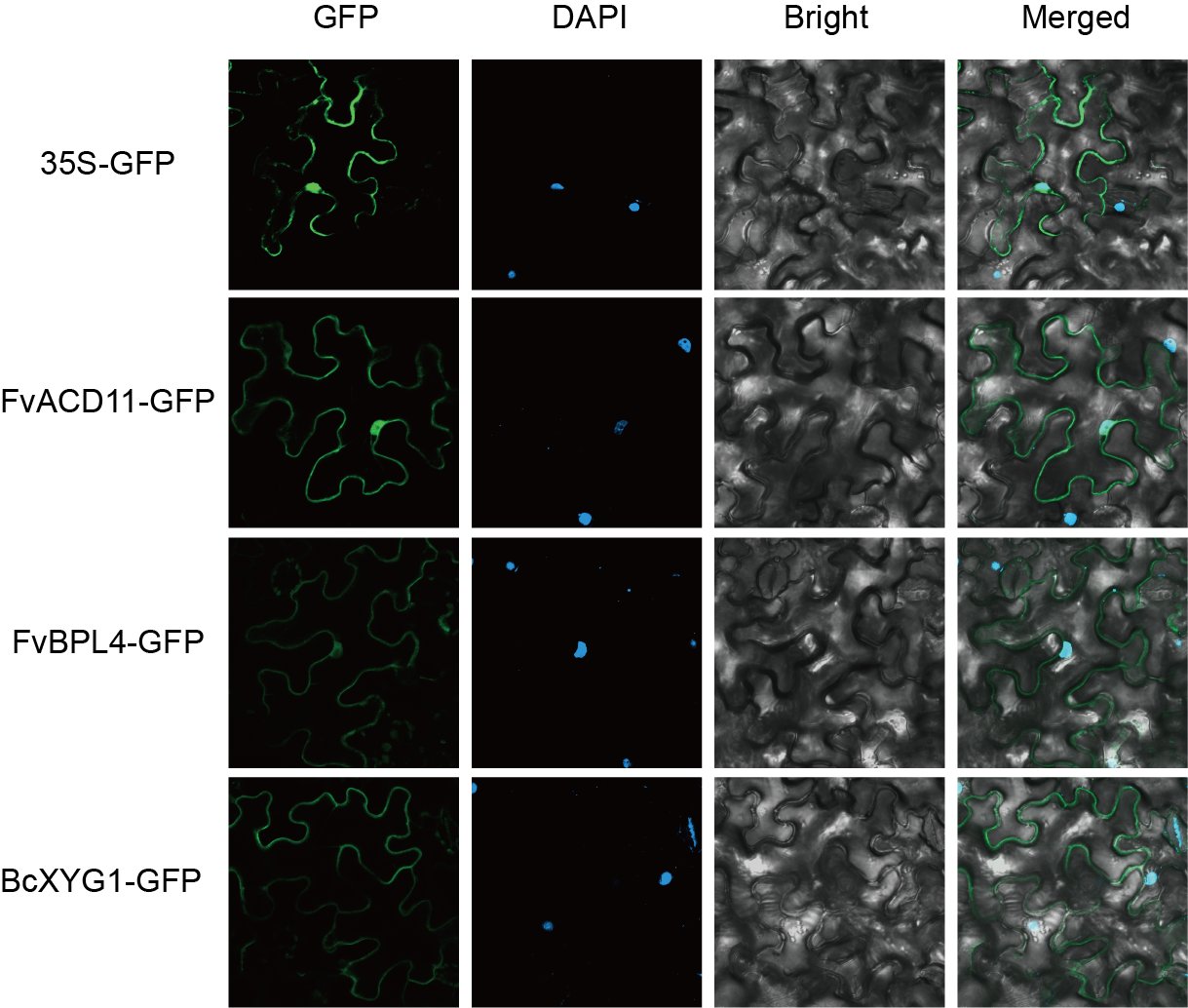


Supplementary figure 4. Subcellular localization of FvACD11, FvBPL4 and BcXYG1 in *Nicotiana benthamiana* leaves. FvACD11-GFP, FvBPL4-GFP and VpBPA1-GFP were transiently expressed in *N. benthamiana* leaves using agroinfiltration.


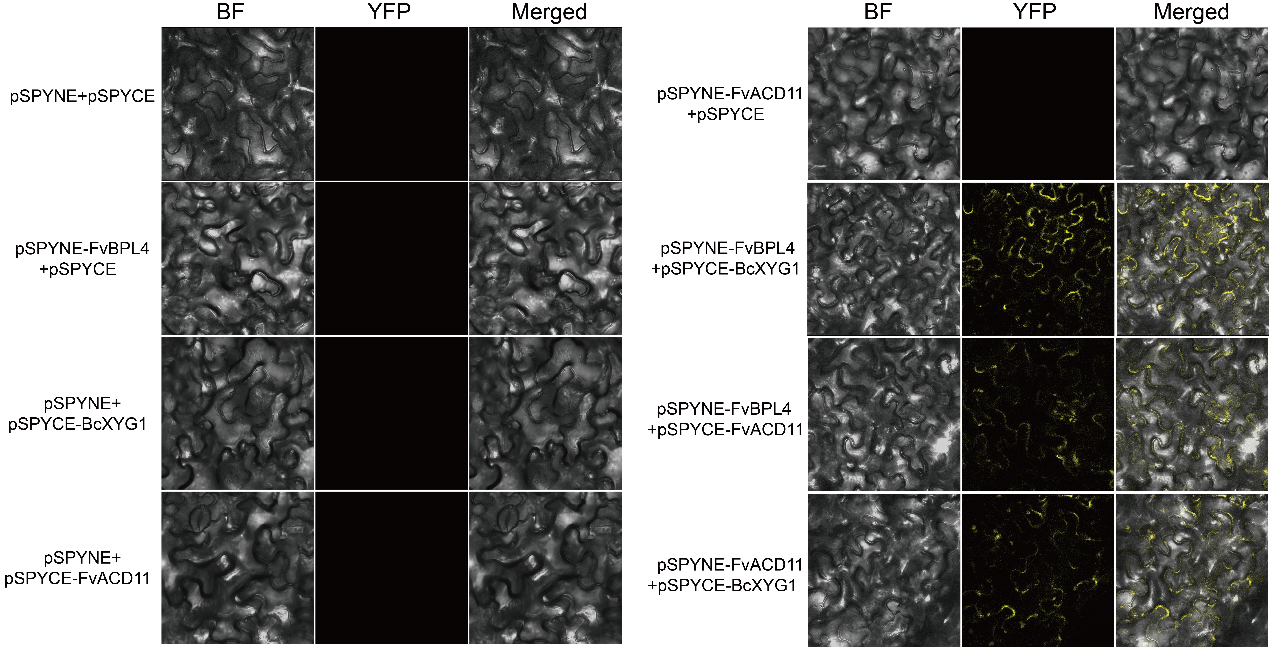


Supplementary figure 5. Verification of the interaction between BcXYG1, FvBPL4 and FvACD11 by BiFC analysis in *N. benthamiana* leaves. The vectors pSPYNE, pSPYCE, pSPYNE-FvBPL4, pSPYNE-FvACD11, pSPYCE-FvACD11 and pSPYCE-BcXYG1 were transiently co-expressed in *N. benthamiana* leaf. Bright-field (BF) and yellow fluorescence (YFP) images were taken using a confocal laser-scanning microscope (514 nm excitation) and merged.
