# Supplementary material for: The Botrytis cinerea effector BcXYG1 suppresses immunity in Fragaria vesca by targeting FvBPL4 and FvACD11
Source: Hortic Res. 2023 Dec 11;11(1):uhad251. doi: 10.1093/hr/uhad251 (PMC10831327; doi:10.1093/hr/uhad251)
Supplement: Web_Material_uhad251 [file web_material_uhad251.zip › Supplementary table.pdf]

Table S1. Primers used in this study

Name of primers	Sequences (5'-3')	Annotation
BPL4-AD-F	CCATGGAGGCCAGTGAATTCATGCAGACGAGAACTGTGCAA	
BPL4-AD-R	AGCTCGAGCTCGATGGATCCTTATGCAGCTATTGGATCCTTTG	
ACD11-AD-F	CCATGGAGGCCAGTGAATTCATGGCGAATGAGAAGCCGCTGAG	
ACD11-AD-R	AGCTCGAGCTCGATGGATCCTTACCAATCTACACCTAAATTCC	Y2H vector construction
XYG1-BD-F	TGGCCATGGAGGCCGAATTCATGAAGTTCACCCAGTCCTTG	
XYG1-BD-R	CGCTGCAGGTCGACGGATCCTTAATTGAGCGAGACGGAGTAGG	
ACD11-BD-F	TGGCCATGGAGGCCGAATTCATGGCGAATGAGAAGCCGCTGAG	
ACD11-BD-R	CGCTGCAGGTCGACGGATCCTTACCAATCTACACCTAAATTCC	
yce-xyg1-F	TGGCGCGCCACTAGTGGATCCATGAAGTTCACCCAGTCCTTG	
yce-xyg1-R	GGTACATCCCGGGAGCGGTACCATTGAGCGAGACGGAGTAGG	
yce-ACD11-F	TGGCGCGCCACTAGTGGATCCATGGCGAATGAGAAGCCGCTG	
yce-ACD11-R	GGTACATCCCGGGAGCGGTACCCCAATCTACACCTAAATTCC	BIFC vector construction
yne-ACD11-R	CTCCATCCCGGGAGCGGTACCCCAATCTACACCTAAATTCC	
yne-BPL4-F	TGGCGCGCCACTAGTGGATCCATGCAGACGAGAACTGTGCAA	
yne-BPL4-R	CTCCATCCCGGGAGCGGTACCTGCAGCTATTGGATCCTTTG	
cLuc-X-F	GTCCCGGGGCGGTACCATGAAGTTCACCCAGTCCTTG	
cLuc-X-R	CGAAAGCTCTGCAGGTCGACATTGAGCGAGACGGAGTAGG	
cLuc-ACD11-F	GTCCCGGGGCGGTACCATGGCGAATGAGAAGCCGCTG	
cLuc-ACD11-R	CGAAAGCTCTGCAGGTCGACCCAATCTACACCTAAATTCC	LCI vector construction
nLuc-BPL4-F	GGGGACGAGCTCGGTACCATGCAGACGAGAACTGTGCAA	
nLuc-BPL4-R	CGCGTACGAGATCTGGTCGACTGCAGCTATTGGATCCTTTG	
nLuc-ACD11-F	GGGGACGAGCTCGGTACCATGGCGAATGAGAAGCCGCTG	
nLuc-ACD11-R	CGCGTACGAGATCTGGTCGACCCAATCTACACCTAAATTCC	
PJX003-X-F	CTCGAGGGGGATCCGAGCTCATGAAGTTCACCCAGTCCTTG	
PJX003-X-R	GGACTAGTCCATGGGGTACCATTGAGCGAGACGGAGTAGG	
PJX003-ACD11-F	CTCGAGGGGGATCCGAGCTCATGGCGAATGAGAAGCCGCTG	
PJX003-ACD11-R	GGACTAGTCCATGGGGTACCCCAATCTACACCTAAATTCC	Subcellular localization vector construction
PJX003-BPL4-F	CTCGAGGGGGATCCGAGCTCATGCAGACGAGAACTGTGCAA	
PJX003-BPL4-R	GGACTAGTCCATGGGGTACCTGCAGCTATTGGATCCTTTG	
001-BPL4-F	AACGATACTCGAGGGGGATCCATGCAGACGAGAACTGTGCAA	
001-BPL4-R	AGTCCATGGGGTACCGTCGACTGCAGCTATTGGATCCTTTGCA	
BPL4-VIGS-F	TGAGTAAGGTTACCGAATTCATGGTGACTAGAGTAAACAGTG	
BPL4-VIGS-R	GTGAGCTCGGTACCGGATCCCAGCACAAACAGCACTTTCCACC	VIGS vector construction
ACD11-VIGS-F	TGAGTAAGGTTACCGAATTCCATTCGAGGAATTTGTTGAGAG	
ACD11-VIGS-R	GTGAGCTCGGTACCGGATCCGAAGAGTTTGTCAATGTACA	
BPL4-1301-F	GAACACGGGGGACTGGTACCATGCAGACGAGAACTGTGCAA	
BPL4-1301-R	AGGTCGACTCTAGAGGATCCTTATGCAGCTATTGGATCCTTTG	
ACD11-1301-F	GAACACGGGGGACTGGTACCATGGCGAATGAGAAGCCGCTG	1301 vector construction
ACD11-1301-R	GGACTAGTCCATGGGGTACCTTACCAATCTACACCTAAATTCC	
XYG1-1301-F	GAACACGGGGGACTGGTACCATGAAGTTCACCCAGTCCTTG	
XYG1-1301-R	AGGTCGACTCTAGAGGATCCTTAATTGAGCGAGACGGAGTAGG	
BPL4-qpcr-F	CAGCGATTGGACAAGATGC	
BPL4-qpcr-R	GATCCTGTATCATTTAGTTTTCTCTC	
ACD11-qpcr-F	TTCGGGTGTTTAGGAATCGC	
ACD11-qpcr-R	CCCTTGGTGACTATAATCTGCTC	
XYG1-qpcr-F	GCCATTACCACTGCCCTTA	qPCR
XYG1-qpcr-R	ATTGACTGTGCTGGAAGCG	
Fv-refe-F	GGGCCAGAAAGATGCTTATGTCGG	
Fv-refe-R	GGGCAACACGAAGCTCATTGTAGA	
Bc-refe-F	CTTAGTACCCCTCAATCGAAT	
Bc-refe-R	GGTATGGTCACTTCCTCCTC	
